# Impaired muscle stem cell function and abnormal myogenesis in acquired myopathies

**DOI:** 10.1042/BSR20220284

**Published:** 2023-01-06

**Authors:** Alyson Deprez, Zakaria Orfi, Lupann Rieger, Nicolas Alexandre Dumont

**Affiliations:** 1 Department of Pharmacology and Physiology, Faculty of Medicine, Université de Montréal, Montreal, QC, Canada; 2 Centre Hospitalier Universitaire (CHU) Sainte-Justine Research Center, Montreal, QC, Canada; 3 School of Rehabilitation, Faculty of Medicine, Université de Montréal, Montreal, QC, Canada

**Keywords:** inflammation, muscle stem cell, myogenesis, myopathies, regeneration, therapeutics

## Abstract

Skeletal muscle possesses a high plasticity and a remarkable regenerative capacity that relies mainly on muscle stem cells (MuSCs). Molecular and cellular components of the MuSC niche, such as immune cells, play key roles to coordinate MuSC function and to orchestrate muscle regeneration. An abnormal infiltration of immune cells and/or imbalance of pro- and anti-inflammatory cytokines could lead to MuSC dysfunctions that could have long lasting effects on muscle function. Different genetic variants were shown to cause muscular dystrophies that intrinsically compromise MuSC function and/or disturb their microenvironment leading to impaired muscle regeneration that contributes to disease progression. Alternatively, many acquired myopathies caused by comorbidities (e.g., cardiopulmonary or kidney diseases), chronic inflammation/infection, or side effects of different drugs can also perturb MuSC function and their microenvironment. The goal of this review is to comprehensively summarize the current knowledge on acquired myopathies and their impact on MuSC function. We further describe potential therapeutic strategies to restore MuSC regenerative capacity.

## Introduction

Skeletal muscles represent 35–45% of an adult body mass, and they are essential for vital functions such as locomotion, postural support, breathing, thermogenesis, and energy homeostasis. It is largely composed by post-mitotic multinucleated fibers that contains the actin-myosin filaments required for muscle contraction. A population of mononuclear cells, called muscle stem cells (MuSCs), or satellite cells, are located between the basal lamina and the sarcolemma of the muscle fibers [1]. During development, myogenic progenitor cells responsible of myofibers formation will give rise to this pool of quiescent MuSCs. These cells are the source of the remarkable regenerative capacity of the skeletal muscle tissue throughout life.

The formation of new muscle tissue (myogenesis) during muscle regeneration is a highly coordinated process regulated by different myogenic regulatory factors (MRFs). Under homeostatic conditions MuSCs are in a quiescent state and express the paired box protein 7 (PAX7), which is indispensable to promote MuSC survival in post-natal muscles [2,3]. After a stimulus such as mechanical stress or growth signals, MuSCs are activated and enter cell cycle to become myoblasts that express the myogenic markers PAX7, and/or myogenic factor 5 (MYF5), and/or myoblast determination protein 1 (MYOD1). After multiple rounds of cell division, myoblasts exit cell cycle to differentiate into myocytes. This differentiation process is accompanied by a reduction in PAX7 expression and an increase in myogenin (MYOG) and myogenic regulatory factor 4 (MRF4) expression. Differentiating myocytes express myomaker and myomerger (also called myomixer or minion) that work independently to regulate the different steps of cell fusion into multinucleated myofiber [4–7]. Myomaker regulates the membrane hemifusion, while myomerger is necessary for fusion pore formation [8]. A portion of these cells will resist differentiation and return to quiescence to maintain the pool of MuSCs for future injury through a complex intrinsic and extrinsic regulatory network, in which the Notch pathway plays a central role [9–12]. Activation of the Notch receptor on MuSCs by differentiated myoblasts or myofibers expressing the Delta-like (DLL) Notch ligands induces the expression of Hes/Hey family members that enhance Pax7 expression and inhibit MyoD and myogenin expression [13]. The expression of specific extracellular components in the MuSC niche, such as collagen-V, collagen-VI, and fibronectin, also provides a microenvironment that supports self-renewal and return to quiescence [14–16].

While MuSCs can restore the muscle structure and function in a matter of weeks to a few months after a severe muscle injury, their regenerative capacity can be diminished in many conditions. Genetic variants in different genes were shown to cause myopathies that impair MuSC function [17]. Mutations in genes such as *PAX7* or *MYMK* (Myomaker) induce primary satellite cell-opathies that affect directly MuSC function and myogenic capacity [18,19]. Mutations in other genes such as *LAMA2* (Laminin 211), *DMD* (dystrophin), or *LMNA* (Lamin A/C) cause secondary satellite cell-opathies that impair both the function of the MuSCs and the muscle fibers. For instance, in Duchenne muscular dystrophy (DMD), the mutation in the *DMD* gene prevents the expression of the dystrophin protein that plays an important role in muscle fiber stability, leading to chronic degeneration, inflammation, and fibrosis [20,21]. This detrimental environment sends conflicting signals that impair the regenerative capacity of MuSCs. The repeated cycles of degeneration and regeneration in DMD overactivate MuSCs, leading to telomere shortening that contributes to the reduction in the MuSC pool overtime [22]. Moreover, lack of dystrophin also directly affects MuSC function by reducing their ability to perform asymmetric cell division that generates self-renewing MuSCs and committed progenitors [23]. Overall, in dystrophic muscles, multiple intrinsic or extrinsic factors contribute to the decline in MuSC function, which impairs the regenerative capacity of the muscles [24–26].

In addition to genetic mutations, other conditions, such as aging, are also known to induce MuSC defects. A decrease in the MuSC pool and a reduction in their regenerative capacity post-injury is observed in aged skeletal muscle. Intrinsic factors such as cellular senescence (state of irreversible cell cycle arrest) was shown to contribute to this MuSC defect in aging [2]. Moreover, extrinsic factors such as chronic inflammation (inflammaging) or hormonal changes also contribute to the exhaustion of MuSC pool and the diminution of their myogenic capacity [27,28]. Notably, physical activity restores the ability of MuSCs to re-enter cell cycle and rejuvenates the regenerative capacity of old muscles [29].

Other conditions such as cancer cachexia can also affect MuSC function and impair their differentiation potential (reviewed in [30]). Paracrine factors secreted in the tumoral environment, such as CXCL1 (C-X-C motif chemokine ligand-1) [31] trigger the nuclear factor κ-B (NF-κB) pathway, which overstimulates Pax7 expression and blocks the expression of the myogenic factors required for myogenic differentiation [32]. Exosomes secreted by cancer cells also activates the Notch pathway that represses myogenic differentiation [33]. Reduction in the expression of ‘anti-inflammatory’ cytokines such as interleukin (IL)-4 and IL-13 in cancer-bearing mice, could also reduce Myomerger expression and impair myogenic differentiation, which can be restored by IL-4 administration [34].

The physiopathology of MuSCs in genetic myopathies, aging, and cancer has been thoroughly investigated in the field, through cellular, animal models, and clinical studies [17,23,30,35–37]. However, how MuSCs are affected in other conditions leading to acquired myopathies is still elusive. In this review, the alteration of MuSCs in respiratory, cardiac, kidney, inflammatory/infectious, and drug-related myopathies will be discussed.

## Respiratory, cardiac, kidney diseases, and myopathies

### Chronic obstructive pulmonary disorder

Chronic obstructive pulmonary disease (COPD) is a group of diseases that can be caused by different conditions such as tobacco smoking or refractory asthma leading to progressive breathing difficulties. This systemic pathology is characterised by increased inflammation of the airways, parenchyma, and pulmonary vasculature that can cause a low-grade systemic inflammation [38]. Dysfunctions in respiratory and limb muscles is a common manifestation of COPD that impact the quality of life of the patients [39]. Oxidative stress [40] and inflammatory state [41] were suggested as a potential causes of muscle weakness and reduction in exercise tolerance [42,43]. Oxidative stress is associated with elevated production of reactive oxygen species (ROS) that activate NF-κB signaling [44], which stimulates the expression of genes such as tumor necrosis factor-α (TNF-α), IL-1β, and IL-6 that promote muscle atrophy in chronic diseases [45,46]. Moreover, NF-kB can directly bind to the promoter region of the muscle-specific ubiquitin ligases MuRF-1 (Muscle RING finger protein-1) and atrogin-1 to promote muscle atrophy [47].

In a mice model of inducible IL-13-driven pulmonary emphysema (IL-13^TG^), that mimics many of the features of COPD, it was shown that there is a reduction in the replication rate and in mRNA expression of *MyoD, Myf5*, and *Myh3* (embryonic myosin heavy chain) in MuSCs cultured *in vitro* [48]. Muscle injury induced by barium chloride injection in these mice results in impaired regeneration and smaller size of newly formed myofibers [48]. MuSCs from IL-13^TG^ mice show an increase in autophagosome accumulation that could be due to impaired autophagosome-lysosome fusion or acceleration of autophagosome generation [48]. Accumulation of autophagosomes has also been observed in muscle biopsies from COPD patients and is correlated with muscle atrophy [49,50]. Although the mechanism behind the accumulation of autophagosome is still elusive, it was shown that a treatment of IL-13^TG^ mice with the autophagy-inducer spermidine rescued the replication rate and myogenesis capacity of MuSCs [48]. Using another transgenic mouse model of pulmonary inflammation induced by the overexpression of the *Tnf*-transgene under the surfactant protein C promoter, it was shown that increased circulating TNF-α levels stimulate local inflammation in the skeletal muscle resulting in muscle wasting and reduction in myoblast proliferation and differentiation in response to physical stress [51]. Consistently, administration of TNF-α in the medium of myoblasts cultured in vitro inhibited their differentiation capacity [51]. In human, the levels of proinflammatory cytokines such as TNF-α and IL-6 are increased in the serum of COPD patients and correlated with muscle wasting [52,53]; however, the expression of these cytokines at the RNA level in the skeletal muscle from COPD patients are similar to healthy individuals [54]. The systemic administration of these proinflammatory cytokines was shown to induce proteolysis and muscle atrophy in preclinical animal models [55,56], but there is no clear evidence of causality between proinflammatory cytokines and skeletal muscle defects in COPD patients.

**Figure 1:**
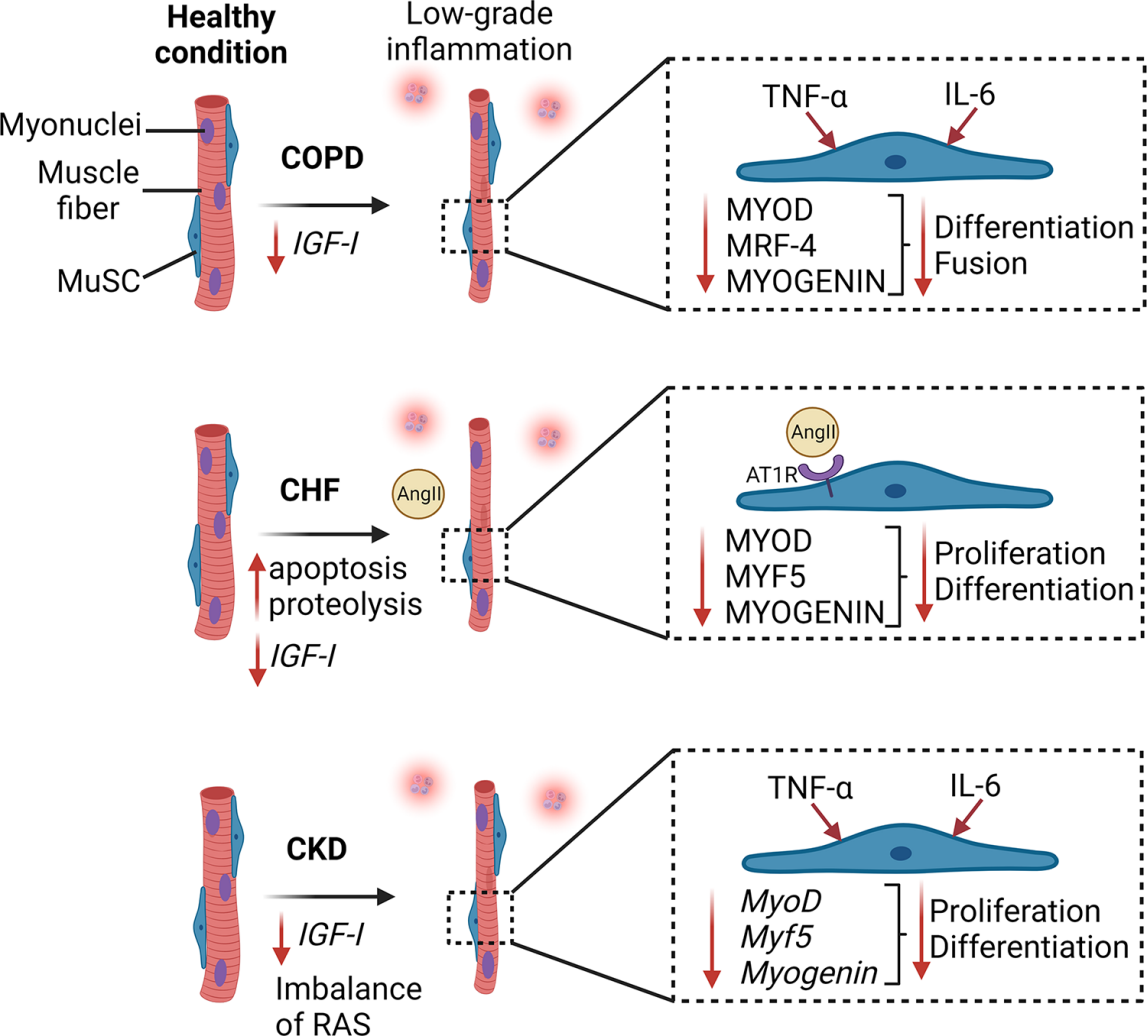
Schematic overview showing the impact of cardiopulmonary and kidney diseases on MuSC function Chronic obstructive pulmonary disease (COPD) induces a decrease in IGF-1 levels in the muscle and a low-grade inflammation characterized by an increase of proinflammatory cytokines (TNF-α and IL-6). These alterations contribute to the decreased expression of MYOD, MRF-4 and MYOGENIN proteins that regulate MuSC activation, proliferation, and differentiation. In chronic heart failure (CHF), low grade inflammation is associated with higher apoptosis and proteolysis, decreased IGF-1 and increased angiotensin II protein levels in skeletal muscle. Angiotensin II acts through AT1 receptor to induce a decrease of MYOD, MYF5 and MYOGENIN in MuSCs. In chronic kidney disease (CKD), reduction of IGF-1 expression, imbalance of the renin-angiotensin system (RAS) and low-grade inflammation (e.g., TNF-α and IL-6) contribute to the reduced expression of *MyoD, Myf5*, and *Myog* genes in MuSC. In these three comorbidities, dysfunction of the MuSCs participates to muscle atrophy and weakness that contribute to the poor prognosis of the diseases.

Analysis of biopsies from the vastus lateralis muscle showed that the number of MuSCs is similar between healthy subjects and COPD patients [57,58]. However, the mRNA expression of *IGF1* (Insulin-like growth factor-1), *MYOD, MYF5* [59] and the protein expression of MYOD [54], MRF4 and MYOG [57] were significantly lower in the vastus lateralis muscle from COPD patients compared with healthy subjects. *In vitro* analysis of MuSCs collected from patients with COPD showed an increase in PAX7 and MYF5 protein expression while myosin heavy chain content was significantly lower during differentiation indicating a reduced ability to fuse and generate mature myotubes [57]. *In vitro* myoblasts from COPD patient showed an impaired capacity to fuse and smaller myotubes diameter compared with those from healthy subject [60]. The proportion of centro-nucleated regenerating fibers is increased in COPD patients that preserved their muscle mass [59], but it is similar to healthy subjects for COPD patients that experienced muscle wasting [57,58], suggesting that in absence of active signs of regeneration COPD patients cannot maintain their muscle mass leading to progressive muscle atrophy. The maintenance of skeletal muscle may be compromised in COPD due to an alteration of their differentiation capacity but also due to premature cellular senescence that can cause an exhaustion of the regenerative potential. *In vitro*, myoblasts from COPD patient exhibit a decrease in maximal telomere length compared with healthy subjects, which is correlated with the cross-sectional area of the thigh muscle [58]. Overall, impairment in MuSCs differentiation/fusion and skeletal muscle regenerative capacity in COPD contributes to the progression of muscle atrophy progression in this population (Figure 1). Adapted physical activity is beneficial for skeletal muscle of COPD patients by decreasing the mRNA and protein levels of myostatin, which is known to inhibit the cell cycle and the differentiation capacity of myogenic cells [61]. Moreover, exercise stimulates the expression of *IGFI* and MYOD [62]. IGF-1 is known to stimulate the expression of MRFs such as MyoD and myogenin and promote myogenic cell proliferation and differentiation [63–65]. Therefore, adapted physical activity in COPD patients could potentially rescue MuSC population and improved muscle regeneration.

### Heart failure

Chronic heart failure (CHF) is a systemic disease characterized by a progressive loss of the systemic perfusion capacity needed to respond to peripheral organs metabolic demands. Patients with CHF present a low-grade inflammation [66], disturbance in the renin–angiotensin system (RAS) [67], reduced exercise tolerance, and skeletal muscle wasting [68,69]. Muscle atrophy and a shift from slow resistant type I fibers to fast fatigable type II fibers [69] are strong predictors of frailty and poor prognosis in CHF individuals [70]. In CHF experimental models (ascending aortic stenosis [69] or monocrotaline intraperitoneal injection [71]), the expression of the myogenic factors *MyoD, Myf5*, and *Myogenin* are down-regulated in peripheral muscles with a reduction of muscle mass and cross-sectional area.

Imbalance in the RAS contributes to the pathogenesis of CHF. In addition to regulating blood pressure and cardiovascular function, the RAS can also regulate skeletal muscle and MuSC function. After a ligation of the left coronary artery in Sprague-Dawley rats, an increased level of angiotensin II, the main effector of the RAS, is responsible for the activation of caspase-3 and down-regulation of *IGFI* in skeletal muscles. These angiotensin II-mediated effects increase muscle proteolysis and apoptosis leading to muscle atrophy [72]. Angiotensin II mediates its effect via 2 receptors, the angiotensin II type I and type II receptors (AT1R and AT2R). After a cardiotoxin injury, in a mice model of CHF (ligation of the left anterior descending artery), MuSC pool and skeletal muscle regenerative capacity are decreased through an imbalance of AT1R and AT2R pathway, characterized by a strong reduction of AT2R expression [73]. A treatment with an antagonist of angiotensin II inhibited muscle wasting and restored the number of MuSCs in rodent model of CHF [74]. Infusion of Angiotensin II during muscle regeneration induced by cardiotoxin injury was shown to inhibit MuSC proliferation/differentiation by acting through the AT1R and Notch-dependent mechanism [74]. On the other hand, stimulation of AT2R activity with an agonist regulates positively MuSC differentiation and muscle regeneration [75]. The opposing effects of the two angiotensin II receptors have been well-described in different conditions [76,77]. AT2R was shown to antagonize AT1R signaling in part by their co-internalization and the inhibition of ERK (extracellular signal-regulated kinase) signaling [78]. To avoid conflicting signals between these signaling axes in MuSCs, there is a temporal regulation of angiotensin II receptors expression at the different stages of myogenesis. AT1R is predominantly expressed in quiescent/activating MuSCs, and AT2R is up-regulated in differentiating myoblasts [74,75].

In vitro culture of human myogenic cells isolated from patients with CHF showed that these cells are able to fuse and form myotubes that express similar levels of myosin heavy chain compared with healthy donors, which suggests that there are no intrinsic dysfunctions in MuSCs, and that it is rather systemic perturbations observed *in vivo*, such as RAS imbalance, that contributes to MuSC defects [79]. Administration of increasing doses of angiotensin II on myoblasts and myotubes *in vitro* were shown to increase the expression of myostatin and the ubiquitin ligases Murf-1 and atrogin-1 [80]. Treatment with the AT1R antagonist losartan ablated this effect. RAS inhibition is associated with a lower prevalence of muscle wasting in CHF patients independently of established risk factors [81] and could be responsible for an alteration of MuSCs that contribute to muscle atrophy (Figure 1).

### Chronic kidney disease

Chronic kidney diseases (CKDs) are a group of diseases characterized by abnormalities in kidney structure or function, which can be caused by different conditions such as hypertension or diabetes. CKD are also associated with impairment of muscle protein synthesis leading to muscle atrophy [82]. Loss of muscle mass of the limbs in CKD is associated with an increased risk of mortality and morbidity [83]. Patients with CKD present impaired IGF-1 signaling, dysregulation of RAS, and systemic inflammation that participate to muscle wasting [72,84]. Similar to CHF, MuSC impairments can also contribute to muscle atrophy in CKD. In CKD experimental model (subtotal nephrectomy on C57BL/6 mice), the number of MuSCs is not affected in the gastrocnemius muscle, but mRNA expression of MRFs is decreased (*MyoD, Myf5, Myog*) [65]. *In vitro*, a reduction in proliferation and differentiation capacity was noted in MuSCs collected from CKD mice compared with control mice [65]. Cardiotoxin-injury in the tibialis anterior muscle leads to a delayed regeneration and smaller newly formed myofibers in CKD mice compared with control. The prolonged and excessive expression of proinflammatory cytokines (e.g., TNF-α and IL-6) in CKD muscle compared with controls during regeneration could contribute to this delayed regeneration [65] (Figure 1). The authors suggest that this degenerative microenvironment in CKD suppresses IGF-1 signaling in MuSCs, which could contribute to the impaired regenerative response. They showed that, similar to CKD mice, IGF-1 receptor knockout mice display muscle atrophy, increased *TGFβ* (transforming growth factor β) expression and muscle fibrosis after muscle injury [65]. Similar to what was observed in COPD patients, the adapted physical activity is beneficial for skeletal muscles of CKD patients by improving the IGF-1 signaling and increasing *MyoD, Myog*, and *Myh3* mRNA expression [82].

## Inflammatory and infectious myopathies

In the skeletal muscle, there is a close interaction between MuSCs and the cellular components of the microenvironmental niche, such as fibro-adipogenic progenitors, blood vessels, and immune cells that collaborate in a well-orchestrated manner to restore a functional muscle tissue after injury [85,86]. Several studies have shown the importance of an organized inflammatory response during muscle injury, in which both innate and adaptive immune cells invade the muscle tissue to coordinate MuSC activation, proliferation, differentiation, and fusion [87–90].

After an injury, there is a release of damage-associated molecular patterns into the extracellular space and activation of the complement system, which trigger a rapid infiltration of the principal components of innate immune cells, such as neutrophils [91]. The activation of neutrophils is a fast process that occurs within minutes after an injury and last up to a few days post-injury [92]. Activated neutrophils clean necrotic cells and debris through phagocytosis in the injured site and amplify the inflammation response by releasing proteases, ROS, and proinflammatory cytokines such as TNF-α, IL-1β, and IL-8 [91–95]. Abnormal accumulation of neutrophils may cause collateral damage to the injured site and impaired the regeneration process [92,96].

After this first wave of immune cell infiltration, neutrophils are quickly replaced by macrophages and T cells. The macrophages are detected at the lesion 12–24 h after injury, and their number keeps increasing significantly during the first few days after injury concomitantly with the rapid decline of the number of neutrophils [87,97]. During the acute phase of regeneration, macrophages adopt a proinflammatory phenotype and play a crucial role in the clearance of cellular debris. Their secretion of several proinflammatory cytokines such as IL-6, TNF-α, and IL-1β contributes to the recruitment of T cells [93,98–100]. Both proinflammatory macrophages and T cells secrete high levels of proinflammatory cytokines that contribute to MuSC activation and proliferation [99,101,102].

After a few of days post-injury, pro-resolving macrophages become the predominant subpopulation in the regenerating muscle [103]. This phenotype switch is triggered by different mechanisms such as the phagocytosis of debris and apoptotic/necrotic cells, and the secretion of anti-inflammatory cytokines by neighboring cells such as regulatory T cells (Treg) [99]. Contrarily to proinflammatory macrophages, the pro-resolving macrophage subpopulation blocks MuSC proliferation and enhances their differentiation and their fusion into myotube [99].

Overall, the communication between the immune system and MuSCs play a central role during muscle regeneration. Controlled expression of proinflammatory and anti-inflammatory cytokines in a timely manner is essential to guide MuSCs through myogenesis after acute injury. Alternatively, dysregulation of the immune system and the chronic expression of proinflammatory signals can directly impact MuSC function and impair the regeneration process, as it is observed in degenerative genetic myopathies such as DMD [104]. In this section, we discuss how autoimmune myopathies or various types of infectious myositis can dysregulate the immune system and impair MuSC function and muscle regeneration.

### Idiopathic inflammatory myopathies

Idiopathic inflammatory myopathies (IIMs) are a spectrum of systemic autoimmune diseases mainly characterised by muscle weakness, elevated levels of serum muscle enzymes, auto-antibodies, and frequently accompanied by extra-muscular manifestations that affect either skin, lung, or joints [105]. The current classification of IIMs suggests the following subgroups: polymyositis, dermatomyositis, immune-mediated necrotising myopathy (IMNM), sporadic inclusion-body myositis (sIBM), clinically amyopathic dermatomyositis, and anti-synthetase syndrome [106–109]. Although these subgroups have overlapping clinical features, the widespread variation in the clinical manifestations of IIMs suggests different pathophysiological mechanisms. Specific human leukocyte antigen haplotypes are among the genetic risk factors for IIMs development [110]. Analysis of high throughput sequencing datasets has identified differentially expressed genes and dysregulated immune-related pathways that highlight the underlying mechanisms of IIMs [111,112]. Several proinflammatory micro-RNAs are up-regulated in IIMs and could contribute to the pathogenesis [113,114]. These different inflammatory signals lead to the chronic and uncontrolled accumulation of immune cells such as CD8+ (cluster of differentiation 8) T cells [115], monocytes [116], and neutrophils [117] that contributes to disease severity. Consequently, the treatment of IIMs is still largely based on anti-inflammatory drugs such as glucocorticoids, antimalarial agents, and immunosuppressive drugs.

**Figure 2:**
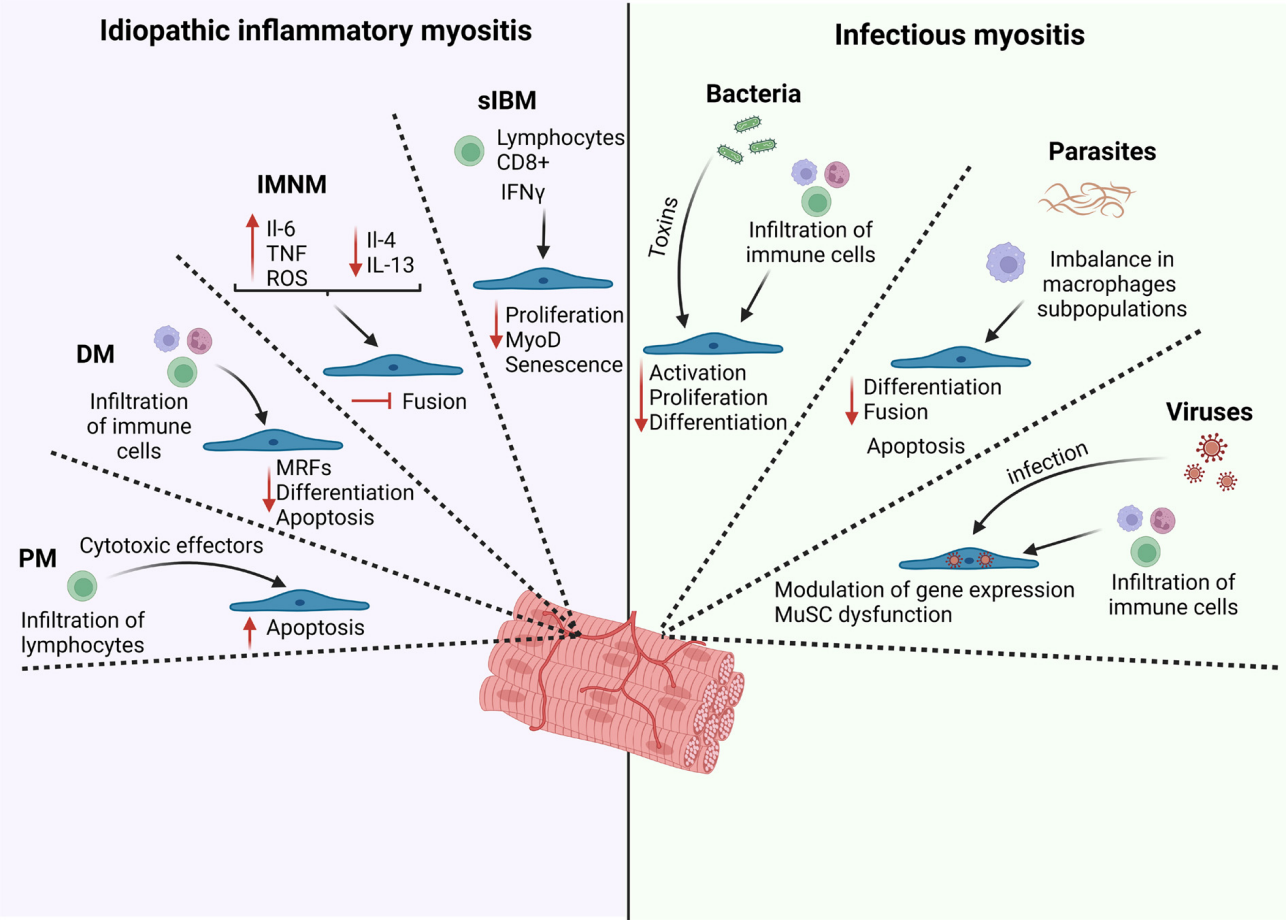
Schematic overview showing the main effects of inflammatory and infectious myopathies on MuSC function Idiopathic inflammatory myositis is characterized by an infiltration of immune cells and/or imbalance of pro- and anti-inflammatory cytokines impairing MuSC function. In both dermatomyositis (DM) and polymyositis (PM) diseases there is an increase in MuSC apoptosis mediated through the excessive accumulation of immune cells. In immune-mediated necrotizing myopathy (IMNM), the MuSC fusion is inhibited due to an imbalance in pro- and anti-inflammatory cytokines. In sporadic inclusion-body myositis (sIBM), paracrine signals from lymphocytes contribute to the reduction in MuSC proliferation and MyoD expression, alongside with MuSC senescence. Infectious myositis are divided in three main causative agents: bacteria, parasites, and viruses. Bacterial infection induces the infiltration of immune cells and/or the secretion of toxins that decrease the activation, proliferation, and differentiation capacity of MuSCs. Parasite infection is characterized by an imbalance of macrophages subpopulation that leads to MuSC apoptosis and lower differentiation and fusion capacity. In viral infection, the infiltration of immune cells and the infection of MuSCs by the viruses modulate the gene expression profile of MuSCs leading to their dysfunction.

The impact of IIMs on skeletal muscle structure and function is well-characterized; however, their effect on muscle regeneration is still elusive. A study comparing the expression of MRFs from muscle biopsies of patients with polymyositis, dermatomyositis, and sIBM showed that there is an increase expression of Pax7, MyoD, Myogenin, and neonatal myosin heavy chain compared with healthy controls. These findings indicate active regeneration in the muscles of IIMs [118]. The next paragraphs describe the current evidence regarding the impact of different IIMs on MuSC function (Figure 2).

#### Polymyositis

Polymyositis is a subgroup of IIMs, characterized by proximal weakness and mild diffuse muscular pain. The histopathological characteristics of polymyositis are mononuclear inflammatory cell infiltration, mainly the CD8+ cytotoxic T cells and CD4+ T cells [119]. Using an *in vitro* model of polymyositis (co-culture of CD8+ T cells with C2C12 myotubes transduced retrovirally with the genes encoding major histocompatibility complex (MHC) class I and a peptide derived from ovalbumin), it was shown that CD8+ T cells invade myotubes and contribute to muscle damage [115].

Analysis of muscle biopsies from polymyositis patients showed that CD8+ T cells are the principal source of necrotic myofibers throughout the expression of cytotoxic effector molecules including perforin 1 and granzyme B [120,121]. Although CD8+ T cells are the predominant immune cells in polymyositis associated with cytotoxicity, it was shown that CD4+ T cells can also cause muscle cell injury directly through Fas cell surface death receptor and its ligand FasL (transmembrane proteins that belong to the TNF family) that are expressed on both infiltrating lymphocytes and muscle fibers on muscle tissue from polymyositis patients [119]. Recently, it was shown in polymyositis patients that the necrosis of muscle fibers is mainly caused by Fas ligand-dependent necroptosis pathway, while MuSC/myoblast apoptosis is triggered by perforin1 and granzyme B [122]. Consequently, the administration of a pan-caspase inhibitor reduced MuSC/myoblast apoptosis but did not affect myotube cell death *in vitro*. Alternatively, the administration of a necroptosis inhibitor (necrostatin-1s) reduced myotube necrosis but did not reduce MuSC/myoblast cell death *in vitro* [122]. Notably, the administration of necrostatin-1s in a murine model of polymyositis did not only reduced myofiber necroptosis but also MuSCs apoptosis (Figure 2) [122]. These results suggest that the reduction of the proinflammatory cytokines and enzymes secreted by necrotic cells and T cells can restore MuSCs function in polymyositis.

#### Dermatomyositis

Dermatomyositis is the most common subtype of IIMs, represented by ∼40% of the total cases. It is characterized by predominant proximal muscle weakness combined with skin rash signs [123]. The histopathological features of dermatomyositis muscle biopsies are characterized by peri-vascular infiltration of immune cells, but less prominent necrosis [124]. Unlike polymyositis, in which CD8+ T cells are the predominant infiltrating immune cells [122], dermatomyositis muscle biopsy is mainly invaded by B cells, macrophages, dendritic cells and CD4+ T cells [125]. Moreover, atrophied myofibers from muscle biopsies of dermatomyositis patients are found in a patchy distribution at specific regions of the fascicle named perifascicular atrophy [124]. These myofibers are characterized by the overexpression of specific proteins around the perifascicular regions, including type I interferon (IFN-I)-induced protein [126], that play a role in the pathophysiology of dermatomyositis [127]. Analysis of muscle biopsies from dermatomyositis patients showed that in the advanced stage of perifascicular atrophy there is a perturbation of MRFs expression, characterized by an increase in Pax7 and Myogenin, but not MyoD [128]. Recently, a study showed that MuSCs isolated from muscle biopsies of patients with dermatomyositis have reduced proliferation and differentiation compared with healthy controls [129]. These cells exhibit higher levels of senescence markers that could partly explain their proliferative defects (Figure 2). Considering that dermatomyositis is associated with sustained inflammation characterized by high levels of IFN-I [126], the authors performed loss- and gain-of-function experiments to demonstrate that high levels of IFN-I decrease the proliferation of MuSCs, while pharmacological inhibition of IFN signaling rescued the proliferation of MuSCs from dermatomyositis patients [129]. These findings suggest that the detrimental effect of dermatomyositis in MuSC function is mediated, at least in part, by paracrine factors.

#### Immune-mediated necrotising myopathy

IMNM is the second largest subtype of IIMs representing ∼20% of the total cases. It is characterized by a severe proximal muscle weakness, low levels of inflammatory cell infiltrate, myofiber necrosis, elevated serum creatine kinase, and limited signs of extra-muscular disease activity [130,131]. Three subtypes of IMNMs are distinguished. Two of them have autoantibodies against signal recognition particle (SRP) or anti-3-hydroxy-3-methylglutaryl-CoA reductase (HMGCR). The third group is a seronegative subtype, in which these autoantibodies cannot be identified [131]. *In vitro*, it was shown that anti-SRP and anti-HMGCR antibodies stimulate myotube atrophy and activation of the ubiquitin E3 ligases MuRF-1 and Atrogin-1 from human muscle biopsies [130]. The fusion of myoblasts into myotubes was also impaired by the treatment with these antibodies (Figure 2). The administration of anti-SRP and anti-HMGCR antibodies is linked to an increased production of proinflammatory molecules (IL-6, TNF and ROS) and a decrease in the expression of anti-inflammatory cytokines (IL-4 and IL-13). Exogenous administration of IL-4 or IL-13 partially rescue the defects in myotubes size induced by anti-SRP and anti-HMGCR antibodies [130].

#### Sporadic inclusion body myositis

sIBM is complex disease caused by an autoimmune component and by degenerative changes characterized by the accumulation of protein aggregates called inclusion bodies [132]. sIBM patients display a slowly progressive muscle atrophy and weakness, signs of fiber damage, and inflammatory infiltrates (mainly cytotoxic CD8+ T cells) with increased IFNγ signature [133,134]. Comparison of MRFs expression from muscle biopsies of patients with different IIMs showed that there is a lower level of MyoD and neonatal myosin heavy chain in sIBM compared with polymyositis [118]. *In vitro* culture of MuSCs isolated from sIBM patients showed that their proliferation is impaired, and the doubling time is longer than normal age-matched controls [135]. However, myoblast differentiation is not impaired *in vitro*. Signs of premature senescence (telomere shortening) and accumulation of cytoplasmic inclusion bodies are also observed in sIBM myoblasts *in vitro*, suggesting intrinsic defects. Recently, a study comparing muscle regeneration in sIBM patients submitted to 12 weeks low-load blood-flow restricted resistance training versus non-exercising sIBM controls, showed that MuSCs content, proliferation, myonuclei number, and myofiber size were not different between groups [136]. This unexpected lack of MuSC response that is usually observed after exercise training suggests that MuSCs are less reactive to growth stimulus or injuries in sIBM (Figure 2).

### Infectious myositis

Infectious myositis is a group of inflammatory myopathies, that is caused by a wide range of micro-organisms including bacterial, parasitic, and viral pathogens [137]. Recently, the various pathogens causing infective myositis and their related clinical features have been comprehensively summarized [138]. Patients diagnosed with infectious myositis may display muscle pain, tenderness, swelling, and weakness. The diagnosis is based on the clinical findings added to laboratory testing, which could be combined to the muscle biopsy findings [138]. The treatments for infectious myopathies depend on the infectious agent. In the next section, we will focus on infectious agents (bacteria, parasites, and viruses) that might impair MuSC function either directly through pathogen–MuSC interaction, or indirectly through the disruption of the immune response.

#### Bacterial infection

The skin protects against bacterial infections by limiting the interaction between the internal and external environment of skeletal muscle. However, bacteria could invade skeletal muscle due to a traumatic injury or to a non-aseptic surgery [138]. Bacterial infection can cause sepsis, a severe inflammatory response, which has been shown to induce muscle atrophy and impair muscle regeneration [139,140]. Single cell RNA sequencing on skeletal muscle in a model of faecal-induced sepsis was shown to induce a rapid immune response and a depletion in the MuSC population that remains at long-term (1-month post-sepsis) [141]. In another model of caecal ligature and puncture, it was shown that sepsis affects MuSC response and their mitochondrial metabolism, leading to impaired muscle regeneration post-injury [139]. *In vitro* experiments showed that the administration of blood serum from septic mice impair MuSC proliferation compared with serum from non-septic mice, suggesting that systemic paracrine factors contribute to MuSC defects induced by sepsis [139] (Figure 2). Various subgroups of pathogenic bacteria, such as gram-positive/gram-negative bacteria, and mycobacteria, can lead to myositis that can affect MuSC function [138].

Pyomyositis is an acute inflammation caused by bacterial infection, which is characterized by neutrophil infiltration into the muscle tissue [142]. Although pyomyositis was commonly observed in the tropical regions, many cases have been detected in temperate regions as well, due to the increase in travel movement [143]. Approximately 90% of pyomyositis cases are caused by *Staphylococcus aureus* (gram-positive), and the other 10% are caused by either *Streptococcus pyogenes* (gram-positive), *pneumococci* (gram-positive), *Salmonella* (gram-negative), or *Escherichia coli* (gram-negative) [144–146]. A study conducted on broiler chicks showed that oral infection of young chicken with *Salmonella Enteritidis* causes systemic infection, and the bacteria could spread out into the muscle tissues [147]. The authors have shown that infected animals have smaller muscle fiber size. Moreover, there is a reduction in the number of nuclei per fiber, which suggest impaired MuSC activity and reduced nuclear accretion to the myofibers. The authors hypothesize bacterial cell wall components (polysaccharides) could directly interact with the Toll-like receptors expressed by MuSCs. Alternatively, an up-regulation in systemic pro-inflammatory cytokines could also impair MuSC myogenic progression.

Myobacteria are another group of bacteria that can affect MuSCs. Particularly, Buruli ulcer is a chronic bacterial infection caused by *Mycobacterium Ulcerans*. While this mycobacterium affects principally the skin, it can also target skeletal muscle to cause weakness. The muscle biopsy following infection is characterized by myofiber atrophy, edema, and the accumulation of connective tissue [138,148]. Muscle histology shows interstitial macrophages and CD4+ T cells around the blood vessels accompanied by myopathic changes. Injection of *M. ulcerans* in the right biceps muscle induces myonecrosis, reduces the size of the myofibers, and the maximal force of the proximate-infected muscles compared with the control mice [148]. *M. Ulcerans* induces its detrimental effect via the release of a toxin called mycolactone. Notably, *M. Ulcerans* or mycolactone administration induces inflammation and myonecrosis, but fails to activate MuSCs, as shown by the absence of upregulation of the MRFs Pax7, MyoD, and Myogenin post-infection [148,149]. The impaired MuSC response could contribute to the delayed regeneration and the accumulation of fibrotic tissue following *M. ulcerans* infection.

#### Parasitic infections

Several parasites can be associated with myositis, such as *Trypanosoma cruzi, Toxocara canis, Schistosoma, Echinococcus, Entamoeba histolytica, sarcocystis*, and others. However, the effect of these parasites on MuSC function has been overlooked. Only *Toxoplasma gondii* (causing Toxoplasmosis), and *Trichinella spiralis* or *pseudospiralis* (causing trichinosis) have been reported to be directly involved in MuSC impairment [138,150] (Figure 2). Each parasite has its unique mode of infection, which triggers a different immune response in the muscle tissue [138].

Toxoplasmosis is a parasitic infectious disease caused by the ingestion of food contaminated with *Toxoplasma gondii*. Although most patients remain asymptomatic, the immunocompromised patients might have severe symptoms. The muscle involvement is characterized by signs of myalgia, weakness, and muscle wasting. Different studies showed that infection with *T. gondii* has a negative impact on both immune cells and MuSCs [151,152]. A first study in mice showed that *T. gondii* induces myofiber necrosis and reduction in muscle strength [152]. The infection leads to the chronic persistence of a proinflammatory state and an impaired capacity of macrophages to adopt a pro-resolving phenotype. Deletion of Treg in the infected mice restores the capacity of macrophages to switch to their pro-resolving state, which was associated with increased signs of muscle regeneration [152]. Cardiotoxin injury to *T. gondii* infected mice leads to reduced MuSC proliferation and exhaustion of the MuSCs pool, which was associated with impaired muscle regeneration (reduced myofiber size) [151]. Single cell RNA sequencing of the infected or uninfected muscle injured or not with cardiotoxin showed that a large proportion of macrophages remains in the inflammatory state post-injury, which is associated with a reduction in the pro-regenerative macrophage subpopulation [151]. Another group has studied the direct effect of *T. gondii* on myoblasts *in vitro* by infecting C2C12 myoblasts with *T. gondii* [153]. Myogenic markers such as MyoD and Myogenin were reduced a few days after the infection. A reduction in myoblast differentiation, fusion, and myotube growth was also observed. These changes were not associated with an increase in cell necrosis or apoptosis. Infected cells also secrete higher levels of pro-inflammatory cytokines such as IL-6 and MCP-1 (monocyte chemoattractant protein-1), and the conditioned medium from infected cells reduces the differentiation of non-infected cells [153]. Overall, *T. gondii* infection impairs MuSC regenerative capacity by targeting MuSCs directly and indirectly through the perturbation of the immune response.

Myositis can be triggered by infection with another type of parasite, the *Trichinella spiralis*. After the intestinal phase, adult worms release newborn larvae into the lymphatic system, which invade the skeletal muscle tissue where they develop and encyst. Muscle invasion causes fever, myalgia, swelling, and muscle weakness. The regenerative capacity of skeletal muscle is severely impeded during *Trichinella* infection, which is linked to mis-differentiation of the MuSCs [154–156]. The larvae invade the myofiber causing the destruction of the myofibrillar organization. Consequently, the MuSCs are activated and proliferate, but they do not fuse to form new myofibers. Instead, they contribute to the development of a specific structure called the nurse cell inside the infected myofiber, which protects the parasite [157]. Transcriptomics analysis of human infected muscle revealed dysregulated expression of genes related to myogenesis, cell proliferation, differentiation, and apoptosis [158,159]. Further analysis revealed that during early infection there is an enrichment in apoptosis-inducing factor-mediated signaling, while only the anti-apoptotic factors survivin and Bcl-2 (B cell lymphoma-2) remains after nurse cell formation [160]. The classical therapy for trichinosis is based on anti-helminthic drugs (e.g., albendazole and mebendazole). It was also showed that mice immunized with gamma-irradiated *Trichinella spiralis* larvae are protected against infection, which leads to reduced muscle damage, Myogenin and Bcl-2 expression [161].

#### Viral infections

There is wide variety of viruses that can cause myositis and different reviews have summarized their main pathological features [138,150,162,163]. Among the viruses known to cause myositis, influenza viruses, coronaviruses, and arboviruses can also impair MuSC function either by directly targeting these cells or indirectly through their impact on the immune system (Figure 2).

Influenza A and B viruses can cause muscle weakness accompanied by fever, pneumonia, and acute respiratory distress syndrome [164]. The exact mechanism of influenza-associated myositis is not yet well described; however, influenza has been isolated from muscle tissues in some cases, suggesting that direct viral invasion into the muscle fibers could contribute to the pathogenesis [165]. *In vitro* experiments showed that influenza A viruses can infect human myotubes and lead to cytotoxic effects; however, myoblasts were partially protected from infection [166]. Influenza infection also results in the systemic release of proinflammatory cytokines such as IL-6, which activates the expression of the ubiquitin E3 ligase Atrogin-1 that promotes muscle atrophy [167]. In the acute phase after the infection, there is an accumulation of macrophages in the skeletal muscle tissue, which is followed by the activation and proliferation of the MuSCs in young mice [167]. However, in aged mice infected with influenza, the macrophage response is blunted, which impairs the expansion of the MuSCs pool [168]. Altogether, these results suggest that influenza infection affects MuSCs indirectly by modulating the immune response.

Severe acute respiratory syndrome coronavirus 2 (SARS-Cov-2) is widely known to induce a variety of symptoms, including myalgia and myositis. SARS-Cov-2 infection can trigger a cytokine-storm characterized by the excessive release of pro-inflammatory cytokines such as IL-1β, IL-6, and TNF-α, which can perturb MuSC function. The infection also increases the expression of ceramides, which are bioactive lipids that can negatively affect myogenesis [169]. SARS-Cov-2 relies on the expression of angiotensin-converting enzyme 2 (ACE2) to invade the host cells. Noteworthy, single cell transcriptomics analysis suggest that *ACE2* is expressed in small specific subsets of MuSCs [170]. However, the direct impact of this coronavirus on MuSCs is still unknown.

Arboviruses are a group of viruses (e.g., dengue virus, West Nile virus, Zika virus, Ross River virus, Chikungunya virus, Mayaro virus, and Sindbis virus) transmitted by arthropods, which can cause fever, rash, polyarthritis, encephalopathy, and myalgia/myositis. Two possible pathophysiological mechanisms of arbovirus-associated myopathies have been described [171]. Like other type of viral infection, the first mechanism is linked to the activation of the inflammatory/immune pathways. Using a murine model infected with alphaviruses (Ross River virus), it was shown that the secretome of macrophages contributes to the development of myositis, and treatment of mice with immunosuppressive drugs prior to infection reduces muscle damage without significantly affecting the viral load in organs [172]. Another study showed that infection with Ross River virus leads to the production of macrophage migration inhibitory factor (MIF), which is associated with muscle damage, and that *MIF*-deficient mice are protected against muscle degeneration despite having similar viral titers [173]. Similarly, the mannose binding lectin pathway, a pattern recognition molecule of the innate immune response, is stimulated after Ross River virus infection, and mice deficient in this pathway are protected against muscle damage without changes in the viral load [174]. The second pathogenic mechanism is linked to the direct infection of MuSCs and myoblasts by arboviruses. Inoculation of Chikungunya virus to human myogenic cells *in vitro* was shown to infect myoblasts but not myotubes. Moreover, immunohistochemistry studies on muscle biopsies from two infected patients revealed that MuSCs were selectively infected by Chikungunya virus, while myofibers were not [175]. Transcriptomic analysis on non-infected versus Chikungunya virus-infected myoblasts have shown altered expression of genes involved in muscular-associated disorders, innate immune responses, cellular growth and death, host metabolism, and virus replication [176]. Similarly, it was demonstrated *in vitro* that human myoblasts could be infected by Zika virus, whereas myotubes are resistant to infection [177,178]. Zika-virus infected myogenic cells undergo a profound modulation of gene expression related to cytokine production, cell death, and immune response [179]. Furthermore, viral replication can also occur within muscle following infection with Ross River virus, Chikungunya virus, Mayaro virus, and Zika virus [171]. Altogether, these findings indicate that viruses can cause MuSC dysfunctions directly through virus cytotoxic effect of infected MuSCs, or indirectly through the perturbation of the inflammatory system response, which in turn has a negative impact on muscle growth and regeneration.

## Toxic myopathies

Many drugs used to treat different diseases or conditions have numerous side effects that can affect skeletal muscle mass or function. These drug-induced myopathies can induce symptoms such as muscle weakness, myalgia, fatigue, and elevation in blood creatine kinase levels [180]. These drugs can also affect the function of MuSCs and muscle regeneration. In this section, we focus on the effect of large classes of drugs such as glucocorticoids and statins, as well as other medications causing toxic myopathies (colchicine, chloroquine, and metformin).

### Glucocorticoids

Glucocorticoids are potent anti-inflammatory molecules that are used for the treatment of various immune-related diseases such as arthritis, allergic reaction, breathing disorders, skin conditions, eyes problem, and muscular dystrophies [181,182]. However, the administration of natural or synthetic glucocorticoids is associated with harmful side effects, such as osteoporosis, cataracts, myalgia and muscle weakness. In skeletal muscle, glucocorticoids activate the members of the ubiquitin-proteasome system, Murf-1 and Atrogin-1, which are associated with muscle protein degradation and atrophy [183]. Moreover, glucocorticoids can also directly target MuSCs and impair myogenesis. Dexamethasone, a synthetic glucocorticoid, was shown to reduce the proliferation and differentiation of myogenic cells *in vitro* [184,185]. Dexamethasone administration in pregnant rat affect the fetal muscle development, leading to reduced myofiber size, MuSC pool, and myogenin expression [186]. Administration of high dose of dexamethasone after muscle injury in adult mice was also shown to impair muscle regeneration and promote heterotopic ossification [187]. Another study showed that it reduces myogenic cell proliferation and the size of the newly formed myofibers [184]. This effect is at least partially mediated by an increase in the expression of myostatin, which reduces the expression of the pro-myogenic gene Akirin 1 and the expression of MyoD, Myf5 and Myogenin. The down-regulation in these MRFs prevents the activation of MuSCs and contributes to muscle atrophy [184]. Additionally, myostatin is known to increase the activity of p21 which inhibits CDK2 (cyclin dependent kinase-2) and prevents the progression of MuSCs from G1 to S phases thus leaving them in a quiescent state [188]. Moreover, activation of the ubiquitin–proteasome system by dexamethasone was also demonstrated to induce the degradation of MyoD in differentiated myotubes [189].

**Figure 3:**
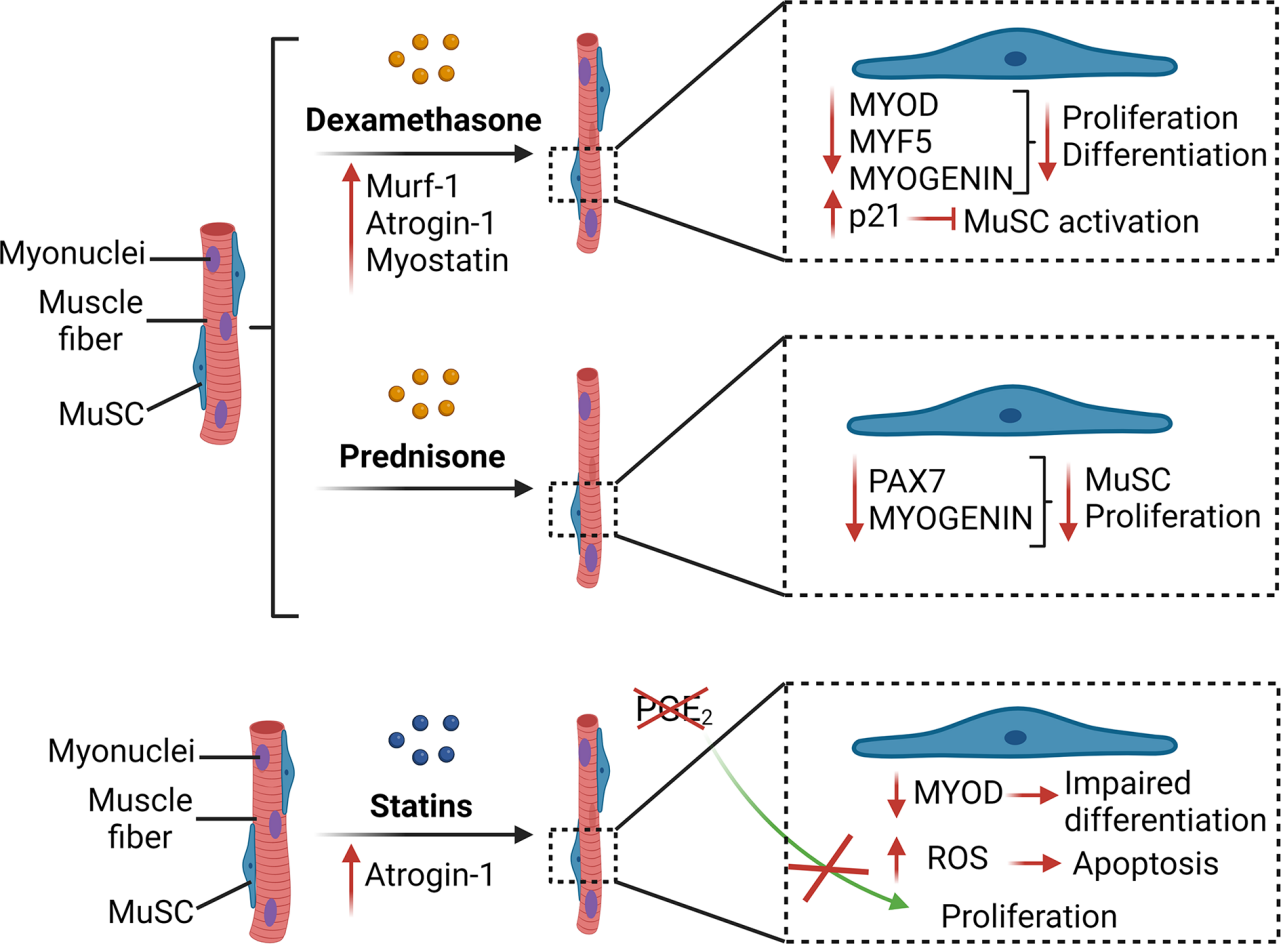
Schematic overview showing the impact of drug-induced myopathies on MuSC function Dexamethasone (glucocorticoid) is associated with an increase in atrophic markers (Murf-1, atrogin-1 and myostatin), a decrease in the expression of MYOD, MYF5 and MYOGENIN, and an increase in the expression of the cell cycle inhibitor p21 that inhibits the activation of MuSCs. Prednisone (glucocorticoid) is associated with a reduction in the expression of PAX7 and MYOGENIN in MuSCs. Statins induce an increase in Atrogin-1 expression and a reduction of PGE2 levels in the muscle, which impair MuSC proliferation. Statins also directly target MuSCs to induce a decrease in MYOD expression and stimulate apoptosis through the release of ROS.

Other types of glucocorticoids also showed similar impact on MuSCs function. Treatment of porcine MuSCs with cortisol increases cytochrome c expression, a major effector in apoptosis, and reduces cell viability [190]. Administration of budesonide, a second-generation glucocorticoid, *in vitro* was shown to reduce Pax7 and MyoD expression and induce a spontaneous differentiation of myoblast cultured in growth medium [191]. Treatment with prednisone on myoblasts *in vitro* reduces cell proliferation and the number of myogenic cells expressing Pax7 or Myogenin [104]. Single myofibers isolated from prednisolone-treated mice show a decrease in the expression of eNOS (endothelial nitric oxide synthase) and nNOS (neuronal nitric oxide synthase) in MuSCs. This decrease in nitric oxide is accompanied by a reduction in the number of MyoD positive cells, which can be rescued by the supplementation of a nitric oxide donor in the culture media [192] (Figure 3).

*In vivo*, the effect of glucocorticoids on muscle regeneration depends on the dosing and timing. Daily administration of prednisone or deflazacort during muscle regeneration of wild-type mice reduces the extent of muscle damage at 7 days post-cardiotoxin injury, but it impairs the recovery of muscle force and physical function at 14 days. At the opposite, weekly administration of these glucocorticoids accelerates the recovery of muscle function [193]. In models of chronic muscle degeneration, such as DMD, the daily or weekly administration of prednisone reduces the excessive inflammation without affecting the number of myogenic cells [104]. Altogether, these findings indicate that glucocorticoids impair the function of MuSCs during acute muscle regeneration, and that the use of these drugs should be controlled and restricted to specific chronic degenerative conditions.

### Statins

Statins are a class of drugs widely used to reduce blood cholesterol levels and are often used in the treatment of atherosclerosis and the prevention of cardiovascular diseases. Muscle pain, fatigue, and weakness are common side effects of the use of statins [194]. These detrimental side effects could be mediated by different mechanisms such as the overexpression of Atrogin-1 and/or mitochondrial dysfunctions [195,196]. In addition, statins can directly impair MuSC function and myogenesis. *In vivo*, treatment of diabetic mice with Fluvastatin decreases the regenerative capacity after cardiotoxin injury, resulting in smaller newly formed myofibers [197]. Similarly, simvastatin decreases the proliferation and differentiation of myogenic cells through the activation of several cellular pathways [198]. Simvastatin inhibits the expression of PTGS1 (prostaglandin-endoperoxide synthase 1) which is an enzyme that biosynthesizes prostaglandins from arachidonic acid. Prostaglandins, such as PGE2 (prostaglandin-E2), contribute to MuSCs proliferation and muscle regeneration [199]. Treatment with specific eicosanoids can partially rescue differentiation in statin-treated myoblasts [198]. Impaired myoblast differentiation by simvastatin was also shown to be mediated by the inhibition of Rac, which can be restored by co-treatment with geranylgeranyl pyrophosphate that participates in post-translational modifications of Rac [199]. Moreover, simvastatin blocks the activation of Akt signalling in myoblasts and myotubes and reduces MyoD expression [200,201]. In addition, simvastatin increases the production of ROS, followed by the release of cytochrome *c* which induces the apoptosis of myoblasts [201]. The increase in miR-1a level that inhibits MAP3K1 (mitogen-activated protein kinase kinase kinase 1) is another simvastatin-activated cascade leading to myoblast apoptosis (Figure 3) [202]. Discrepancies are observed in the literature regarding the susceptibility to statin-induced cytotoxic effect in the different stages of myogenesis. Some studies show that myoblasts are more affected than myotubes, while others indicate the opposite, which could depend on the outcome measured [200,201].

### Other medications

In addition to glucocorticoids and statins, other types of drugs are also known to cause toxic myopathies that impair the proper functioning of MuSCs.

Colchicine, a drug used to reduce inflammation to treat gout and other inflammatory disorders, is also associated with toxic myopathies, particularly when combined with other medications such as statins [203]. Combination of colchicine and statins simultaneously activates autophagy signaling and inhibits the degradation of the newly formed autophagosome, leading to myotoxicity [204]. Administration of colchicine in young and old mice was shown to impair autophagy resulting in dysfunctions in skeletal muscle mitochondria [205]. *In vitro*, the addition of colchicine blocked cell division in myoblasts and led to myotube fragmentation [206]. In different models of muscle regeneration, the administration of colchicine was shown to block myogenesis and the formation of new myotubes, especially when administered during the peak of cell division around 3 days post-injury [207,208].

Chloroquine, an anti-malarian drug, has also been associated with toxic myopathies [209]. Treatment of C2C12 myoblasts with chloroquine showed a dose-dependent reduction in autophagy [210]. Blocking autophagy with chloroquine in single myofibers or freshly sorted MuSCs *in vitro* inhibits MuSC activation and cell cycle entry [211]. Treatment of 3D muscle construct in vitro with increasing doses of chloroquine showed a reduction in contractile protein expression and tetanus force of the muscle tissue [212].

Metformin, a hypoglycemic agent used in the treatment of Type 2 diabetes can also affect MuSC function. Treatment of single myofibers with metformin delays the activation of MuSCs and their cell cycle entry [213]. Moreover, administration of metformin to myoblast cultured *in vitro* delays their differentiation and the formation of myotubes. Metformin inhibits the phosphorylation of ribosomal protein S6 in MuSCs, which could explain part of the phenotype observed. *In vivo*, the administration of metformin during muscle regeneration post-cardiotoxin injury reduced the number of centro-nucleated fibers and the size of the newly formed fibers [213].

Altogether, these findings show that glucocorticoids, statins, and others class of drugs impair the function of MuSCs. Moreover, interactions between certain drugs accentuate the negative effects observed on MuSCs [214,215].

## Therapeutics avenues

As described in the previous sections, acquired myopathies can impair MuSC function through different direct and indirect mechanisms (Figures 1-3). In consequences, the optimal therapeutic approach will vary depending on the causative agent (e.g., comorbidities, inflammation/infection, or side effect of drugs). Novel insights on the underlying mechanisms of MuSC defects open new therapeutics avenues that could alleviate the muscular symptoms in acquired myopathies.

Adapted physical activity is considered one of the most effective strategies to counteract muscle atrophy and impaired muscle regeneration related to chronic inflammatory diseases [216]. Regular physical activity is known to reduce inflammation [217,218], increase the expression of genes related to myofibers metabolism and MuSC function [219,220], and expand the MuSC pool [221–223]. Physical activity was also found to be beneficial in autoimmune myositis [224–226]. Physical exercise was also shown to reduce some of the muscular symptoms associated with statins [227]; however, the impact on MuSC is unknown.

Another dysregulated process that is a common feature of many acquired myopathies is the overproduction of ROS. Antioxidants were tested as another strategy to enhance MuSC function and muscle regeneration in acquired myopathies. Vitamin E supplementation can modulate inflammation [228] and improve antioxidant defenses [229], protein synthesis, and myogenic markers in skeletal muscle [230]. However, antioxidant treatment such as vitamins C and E were unsuccessful to restore muscle function in COPD patients [231,232]. Nonetheless, antioxidant therapies could be used in combination with other approaches such as adapted physical activity to improve the impact on muscle recovery in patients with COPD [233].

Chronic inflammation is also a hallmark of many myopathies, and consequently, anti-inflammatory drugs were investigated as a therapeutic approach for acquired myopathies. As discussed above, while glucocorticoids are one of the most widely used anti-inflammatory drugs, they can also impair MuSC function and stimulate muscle atrophy. Therefore, other molecules have been explored such as neutralizing antibodies or antagonists targeting specific cytokines or their receptors. For instance, anti-TNF-α therapies such as infliximab reduced inflammation and enhanced muscle regeneration post-injury [234]. However, anti-TNF-α therapies led to disappointing results in clinical trials for diseases such as COPD, heart failure, and polymyositis/dermatomyositis [235–238]. Further investigation with novel TNF-α inhibitors (e.g., etanercept), or drugs targeting other cytokines such as IL-1 (e.g., anakinra) or IL-6 (tocilizumab) are ongoing [239]. Nevertheless, it is possible that the inhibition of only one cytokine will not be sufficient to overcome the chronic and complex inflammatory process in acquired myopathies [240]. Other medication targeting upstream inflammatory signaling pathways, such as NF-kB or p38MAPK inhibitors, are currently investigated in clinical trials [241]. Overactivation of the NF-kB pathway in MuSCs from a mouse model of DMD was shown to contribute to the exhaustion of the MuSC pool and to the impaired muscle regenerative capacity[242]. Consequently, NF-kB inhibition was shown to enhance muscle function in mouse and dog models of DMD [243]. Recently, a new class of bioactive lipids derived from omega-3 fatty acids, named resolvins, has shown promise for different immune-related diseases. Resolvin-D2 was shown to enhance muscle function compared to glucocorticoids by targeting inflammation and rescuing MuSC regenerative capacity in a mouse model of DMD [104]. Resolvin-D1 was shown to reduce inflammation and oxidative stress in a model of COPD [244]. The impact of pro-resolving bioactive lipids on diseases such as asthma is currently investigated in clinical trials [245].

In addition to pharmaceutical molecules, the therapeutic potential of cellular therapies has also been explored for acquired myopathies [239]. Injection of mesenchymal stem cells in the muscle of mice submitted to a caecal ligature and puncture model of sepsis, was shown to reduce proinflammatory signals, restore the mitochondrial and metabolic function of MuSCs and their regenerative potential, resulting in enhanced muscle strength [139]. Case reports have shown that the transplantation of autologous stem cells or allogenic mesenchymal stem cells could alleviate the symptoms and enhance muscle strength in refractory cases of myositis [246–249]. However, high quality randomized controlled trials are needed to fully delineate the therapeutic potential of cell transplantation for the treatment of acquired myopathies.

## Conclusion

MuSCs are the cellular protagonists providing the remarkable regenerative capacity of skeletal muscle. Their activity is closely regulated by molecular and cellular components of their niche. Disturbance in the fine balance between MuSCs and their microenvironment can affect their ability to activate, proliferate, differentiate, and fuse to restore tissue integrity and function. Different pathological conditions (e.g., comorbidities, infections, or chronic drug exposure) are associated with the development of acquired myopathies. In this review, we demonstrated that many acquired myopathies trigger inflammatory mechanisms that are responsible of an imbalance in the microenvironment of MuSCs leading to an alteration of their function. Alternatively, the causal agents of these acquired myopathies can also directly target MuSCs to affect their expression of MRFs and impair their function and cell fate decision. MuSC defects in acquired myopathies could contribute to muscle atrophy/weakness and to the progression of the diseases. Considering the crucial role of MuSCs in muscle growth and regeneration, it is important to understand the underlying mechanisms leading to MuSC defects, in order to develop new therapeutic avenues to rescue MuSC function and alleviate the progression of the symptoms in acquired myopathies.

## Abbreviations

ACE2: angiotensin-converting enzyme 2
CDK2: cyclin dependent kinase-2
CHF: chronic heart failure
CKD: chronic kidney disease
DMD: Duchenne muscular dystrophy
HMGCR: 3-hydroxy-3-methylglutaryl-CoA reductase
IFN-I: type I interferon
MIF: macrophage migration inhibitory factor
MRF: myogenic regulatory factor
MuSC: muscle stem cell
MYF5: myogenic factor 5
MYOD1: myoblast determination protein 1
MYOG: myogenin
PAX7: paired box protein 7
PGE2: prostaglandin-E2
PTGS1: prostaglandin-endoperoxide synthase 1
ROS: reactive oxygen species
SARS-Cov-2: severe acute respiratory syndrome coronavirus 2
SRP: signal recognition particle
